# Food Protein Nanofibril Gels: From Conditions, Types and Properties to Applications

**DOI:** 10.3390/foods13142173

**Published:** 2024-07-09

**Authors:** Chen Guan, Changyuan Wang, Shixin Fu

**Affiliations:** 1 College of Food Science, Heilongjiang Bayi Agricultural University, Daqing 163319, China; 2 Quality Supervising and Testing Center of Ministry of Agriculture and Rural Affairs for Agricultural Products and Processed Goods, Daqing 163319, China; 3 National Coarse Cereals Engineering Research Center, Heilongjiang Bayi Agricultural University, Daqing 163319, China; 4 Institute of Animal Science and Technology, Heilongjiang Bayi Agricultural University, Daqing 163319, China

**Keywords:** nanofibril, self-assembly, hybrid gels, aerogels

## Abstract

Many food proteins can be assembled into nanofibrils under pH conditions far from the isoelectric point and with a low ionic strength by heating them for a long period. These food protein nanofibrils (FPN) have outstanding functional and biological properties and are considered sustainable biomaterials in many fields. In this study, we review the recent developments in FPN gels and introduce the key factors in promoting food protein self-assembly in order to create functional gels. The major variables discussed are the morphology of nanofibrils, protein concentration, heating time, and the type and concentration of salts. We also highlight current advances in the formation and properties of different types of FPN gels. In addition, the various applications of FPN gels in bioactive and nutrient delivery, adsorbents for CO_2_ and toxic pollutants, cell scaffolding biomaterials, biosensors, and others are introduced and discussed.

## 1. Introduction

Protein has attracted much attention because of its nutritional value and important biological, physical, and chemical functions. The physical and chemical properties of protein-based systems are regulated by pH value, temperature, and ionic strength [1]. Under acidic conditions (far from the isoelectric point) and low ionic strength, many food proteins can be assembled into nanofibrils by heating them for a long period [2]. 

Many studies focus on the formation conditions, properties, and applications of food protein nanofibrils (FPN) [3,4,5]. Few publications have comprehensively discussed the recent developments of FPN gels. In this review, the factors affecting the formation of FPN gels are discussed. Different types of FPN gels and their properties are then summarized. Finally, the various applications of FPN gels are introduced in this article. Overall, we hope this review will inspire researchers to explore the potential of FPN gels further.

## 2. FPN, Concept, Specification, and Reactivity

Self-assembled nanofibrils, also known as amyloid proteins, can be obtained from different protein sources (i.e., animals, plants, and microbes) [3]. Animal proteins, such as milk proteins [4,6,7,8], blood proteins [9], and hen egg-white proteins [10,11], can self-assemble into nanofibrils under appropriate conditions. Plant proteins [12], including cereal proteins (wheat [13], oat [14], and rice bran albumin [15]), legume proteins [16] (soybean [17], mung bean [18], pea [19], black bean [20], and faba bean [21]), tuber proteins (potato [22]), seed proteins (rapeseed [23], cottonseed [22], peanut [24], sunflower seed [25], hemp seed [26], and buckwheat seed [27]), and pseudocereal proteins (amaranth [28]), can also form nanofibrils. Some microbial proteins, such as filamentous fungi [29] and curli [30], can cause fibrillation.

Nanofibrils are slender and usually consist of two or more spirally arranged precursors with a diameter of approximately 2–10 nm and a length of usually more than 1 μm. When examining them via X-ray diffraction (XRD) and Fourier-transform infrared (FTIR) spectroscopy or circular dichroism (CD) spectroscopy, the nanofibrils mainly exhibit a β-sheet-rich secondary structure and a characteristic cross-β structure [31].

Compared with native proteins, food protein nanofibrils have a high aspect ratio, good biocompatibility, and non-toxic properties [32]. Hence, food protein nanofibrils are suitable as thickeners, gelling agents, foam stabilizers, etc. [3,33], and have potential application prospects in different research fields such as food science, biomedicine, and nanotechnology [34]. 

Gelation is one of the most important functional properties of food protein, as protein gels can trap or immobilize water, fat, and flavor substances. The formation of the gel begins with the denaturation (unfolding) of protein molecules via heating, followed by protein aggregation to form a 3D gel network [35]. At a neutral pH, the low net charge of the protein produces a smaller repulsive force, and the protein molecules randomly aggregate through hydrophobic interactions, disulfide bonds, and hydrogen bonds to form opaque granular gels [36]. When the pH deviates from the isoelectric point, protein molecules demonstrate a high-charge self-assembly to form nanofibrils. Owing to the balance of hydrophobicity and hydrophilicity, tangled fibrils further form denser fibril gel networks. The elasticity, water-holding capacity [37], rheological properties [38], and homogeneity [39] of nanofibril gels are higher than those of conventional protein gels. Thus, food protein nanofibril gels might be more suitable to deliver bioactive ingredients [40,41], water purification [42], CO_2_ captures [20], and cell cultures [43]. However, the ability to form the gels and the mechanical strength of the gels prepared with diverse FPN are different. 

## 3. Factors Affecting FPN Gelling

Food proteins can convert into nanofibrils under different conditions [3,42,44]. The protein concentration and high temperatures play a role in protein fibril self-assembly. Some proteins should be fabricated at 80–90 °C for a long time to form fibril structures [12]. Further, the protein concentration can affect the morphology and β-sheet content of fibrils. The pH value affects the cleavage of peptide bonds and regulates electrostatic interactions [2]. The ionic type and strength can also modulate the electrostatic interactions between the protein chains [45]. Shearing and high pressure affect the kinetic process and the morphology of FPN. These factors finally affect the properties of the FPN gel.

### 3.1. The Morphology of the Nanofibrils

Herneke et al. [12] and Li et al. [46] produced nanofibrils from different food proteins and found that the morphology of the nanofibrils with a curly or straight shape depended on different food sources. Rigid fibrils prepared using chickpeas, lentils, and pumpkin seeds could not form gel networks through ion-induced gelation. Meanwhile, flexible fibrils prepared using kidney beans, black beans, cowpeas, and mung beans could form a gel during ion induction. Black bean nanofibrils were short and curly, and the mesh size of black bean nanofibril gels was larger than that of the other samples. The aerogel was not conducive to amine modification and had a weak adsorption capacity for carbon dioxide [20]. Munialo et al. [47] showed that pea protein fibrils with a highly flexible, curly morphology and lower degree of alignment formed a lower-strength gel than semi-flexible whey protein isolate (WPI) fibrils. In contrast, Li et al. [46] demonstrated that the networks of plant protein nanofibril gels were more extensive than those of *β*-Lactoglobulin (*β*-lg) nanofibrils, which might be related to a higher content of flexible fibrils in plant proteins.

### 3.2. Protein Concentration

The protein and peptide concentration influence the fibril network and gelation properties. Nanofibril gels must be formed at sufficient concentrations to promote gelation. The increase in the fibril concentration first leads to a phase transition from the isotropic phase to the nematic phase, and a further increase leads to the formation of the gel phase [48]. A higher concentration benefits β-sheet contents and the crosslinking degree of protein. Thus, the mechanical strength of gel networks can be regulated by the concentration of nanofibrils [49]. The critical gelation concentration of β-lg nanofibril was 6.8% at a pH of 2 and a low ionic strength [50]. Bolder et al. [51] found that when the concentration of whey protein nanofibril was higher than 6 wt%, a strong gel was formed. Xu et al. [52] found that a soy protein isolate (SPI) fibril gel was formed after 24 h at a concentration of 30 g/L. At a concentration of 50 g/L, self-supporting hydrogels were observed to form at 12 h. At a higher protein concentration, the elastic modulus (G′) and loss modulus (G″) of the fibril solution increased significantly, forming a denser network structure. Although at protein concentrations as high as 160 mg/mL, pea protein fibrils cannot form a self-supportive gel, which may be due to the difference in flexibility between pea and whey protein fibrils [47]. Lentil protein nanofibrils could form a gel with a critical protein concentration of 1.5% at pH 2 and 16 h heating. Nevertheless, the gelation time decreased to 1–2 h when the concentration was 8–10% [38]. The water-holding capacity (WHC) and gel strength increased with the increase in the lentil protein nanofibril concentration, which may be due to the smaller and more uniform pore size of the gel with higher nanofibril concentration. Furthermore, the critical protein concentration of cold-set nanofibril gels is much lower than that of heat-induced gels [53].

### 3.3. Heating Time

When the heating time is increased, the protein molecules may undergo full unfolding to expose the active groups, which then interact with each other to form a homogeneous and interconnected gel network. Thus, exposing the lentil protein isolate (LPI) nanofibril to a long heating time contributed to an increase in light transmittance, WHC, and gel strength of lentil protein nanofibril gel. On the other hand, a longer heating time resulted in less elasticity and lower fracture strain of the gel due to increased protein-protein interactions [38]. Zhang et al. [15] found that the mature rice bran protein fibril formed due to heating was also significantly related to the gel properties. As more mature fibrils were added to the solution, the gel strength of rice bran protein increased. Thermosonication treatment showed a shorter time to induce self-supporting Faba bean protein fibril gels than the conventional heat treatment [21].

It is important to note that longer heating times and higher incubation temperatures during the formation of nanofibril gels might lead to the formation of undesirable colors, where color formation is caused by the Maillard reaction [54] and protein oxidation [55,56]. This extensive darkening might be undesirable in food products containing FPN or lead to health-related hazards.

### 3.4. Ionic Type and Strength

The strength and morphology of fibrils are influenced by the ionic strength and type, which in turn influence the macroscopic properties and the strength of fibril gels. Ye et al. [56] demonstrated that adding different salts to coordinate with water molecules before incubation was the key to the growth of WPI nanofibrils (WPF). In contrast, added ions after the growth of the fibrils could not support the formation of gels. It was also demonstrated that the strengths of fibril hydrogels with divalent Ni^2+^, Mg^2+^, and Co^2+^ were greater than when adding monovalent Na^+^ and K^+^. However, the iron-containing fibril samples failed to form hydrogels regardless of the iron concentration, suggesting that iron may interact with carboxylic acids on the protein chains. Similarly, Mohammadian et al. [57] demonstrated that divalent cation type has an important effect on the texture and WHC of WPF gels. The gel strength increased with the decrease in the ionic radius (in the order of Zn^2+^ > Mn^2+^ > Ca^2+^). The larger the ionic radius, the lower the surface charge density, so a diminished interaction between protein molecules is established by counter ions. Khalesi et al. [58] adjusted the rheological properties of WPI gel by adding different lengths of WPF at various NaCl concentrations. Adding NaCl to both the short-fibril and long-fibril-containing hydrogel led to an increase in the elasticity of gels. The G′ value increased with NaCl concentrations and peaked at 100 mM of NaCl. G′ of a WPI fibril-gliadin nanoparticles (WPF-GNP) hybrid hydrogel could be enhanced with an increase in the NaCl concentration [59], which was attributed to the decreased electrostatic repulsion and the increased electrostatic interaction between the protein chains due to screening charges. Furthermore, the formation time of the fibril hydrogel was reduced when a high concentration of CaCl_2_ was used [60].

## 4. Preparation of Different Types of FPN Gels

**Figure 1:**
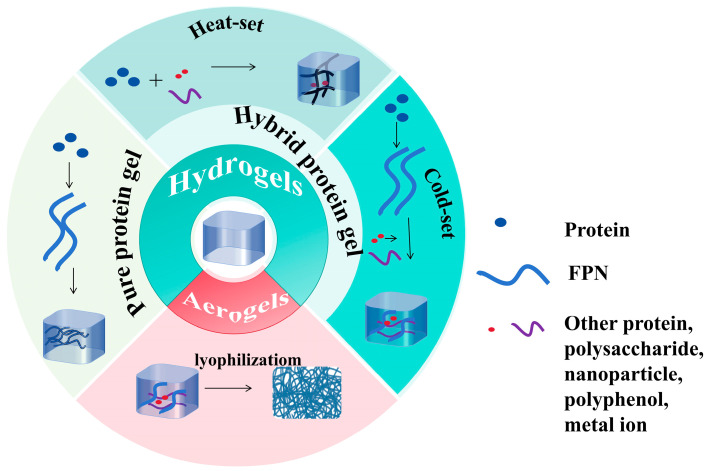
Different types of FPN gels.

FPN gels can be divided into many types according to different production methods and are shown in Figure 1.

### 4.1. Pure Protein Nanofibril Gels

A pure nanofibril gel can be prepared from many food proteins at a higher temperature and protein concentration. A lentil protein nanofibril could form a translucent and highly homogeneous gel after being heated at 90 °C for different incubation times [38]. Ovalbumin samples formed a high water retention gel under heating at 90 °C for 60 min [61]. 

The gel strength increased with an increase in the WPF concentration in the WPI solution, especially after adding 1% WPF, where the elastic modulus of the gel increased by 10.6 times. The increase in the fibril concentration promoted the interaction between the fibrils [62]. Similarly, the gel strength and microstructure of the rice bran albumin gel could be significantly increased via the addition of rice bran albumin nanofibrils [15]. Faba bean protein fibrils formed a viscoelastic network under thermosonication treatment and showed thermoreversible gelation behavior [21].

### 4.2. Hybrid Protein Nanofibril Gels

#### 4.2.1. Hot-Set Nanofibril Gels

Under heating, the gel properties or functional properties of nanofibrils can be regulated by crosslinking with other biopolymers, such as other proteins, polysaccharides, nanoparticles, polyphenols, and metal ions. 

Adding panda bean protein fibrils to pea protein isolate (PPI) can improve the strength and elasticity of the gel because of the enhanced hydrogen bonds and hydrophobic interactions [63]. Cellulose nanocrystals (CNCs) can bind with soy protein isolate amyloid fibrils through hydrogen bonds and improve the gel quality [64].

Hybrids of WPF and GNP produced a WPF-GNP hydrogel at a low protein concentration of 2%wt and 3 h heat treatment (85 °C water bath) [59]. The helical ribbons of nanofibrils could bond with GNP to form crosslinked structures and then facilitate hydrogel formation. The viscoelasticity of the WPF-GNP hydrogel could be regulated by the pH, ionic strength, and protein ratio. The elastic modulus of WPF-GNP hydrogel was maximized at a pH of 4.0, which was higher than that of WPI-GNP hydrogel. The addition of Epigallocatechin gallate (EGCG) shortened the heating time for the ovalbumin to form a gel at 80 °C and a pH of 3 [61]. Hydrophobic interactions, hydrogen bonding, and van der Waals forces were the main interactions between EGCG and ovalbumin. G′ increased with the increase in the EGCG concentration, especially at 5 μM/g of EGCG. Heat-induced gels could also be induced by metal ions. Ye et al. [56] confirmed that adding different metal ions before incubation at 90 °C for 24 h induced the formation of a WPF gel. Similarly, a hybrid WPF gel could be prepared by mixing with CaCl_2_ and heating at 80 °C for 30 min. Hydrophobic interactions and hydrogen bonds played a crucial role in the formation process of the gel [65]. Bolisetty et al. [48] found that *β*-lg nanofibril gels were induced by NaCl salt at a pH of 2 after the heat treatment and a temperature of 90 °C.

#### 4.2.2. Cold-Set Nanofibril Gels

Recently, applying FPN to prepare cold-set gel has become a research hotspot. FPNs are produced by first using the thermal-acid method. After cooling, gelation is induced by adding crosslinker agents at low temperatures [53,57]. The structure of cold-set nanofibril gels is mainly stabilized by hydrophobic interaction, van der Waals interaction, and intermolecular disulfide bonds. The critical protein concentration of cold-set nanofibril gels is much lower than that of heat-induced gels or conventional cold-set gels [53]. Veerman et al. [53] reported that the critical concentration of a whey protein nanofibrils cold-induced gel was as low as 0.3%.

Several crosslinker agents can form a cold-set protein hydrogel, such as salts [48], acids [66], and enzymatic and chemical treatment. Hybrid gels based on different crosslinkers exhibited different microstructures and properties. During gel formation, the pore size was narrowed by adding poly(ethylene glycol) (PEG) to the hen egg-white lysozyme (HEWL) nanofibril and increased by adding gelatin [60]. Different crosslinking agents also affect the incubation temperature and incubation time of a hybrid HEWL nanofibril hydrogel [60]. The samples with no crosslinker successfully formed gels when heated at 37 °C. Meanwhile, adding gelatin to the HEWL nanofibril solution supported a rapid gelation rate when kept at room temperature.

A Ca^2+^-induced bovine serum albumin/*β*-lg fibril cold-set hydrogel showed an excellent ability to absorb energy and release stress [67]. Plant protein fibrils from kidney beans, black beans, cowpeas, mung beans, and oats can form gel networks by dialyzing with the NaCl solution for 2 days [46,68]. The divalent cation type significantly affected the texture and properties of the whey protein nanofibril gel [57]. The zinc-induced fibril hydrogel was firmer and had a higher WHC and tighter gel structure than calcium- and manganese-induced gels. 

Cold gelation of WPI fibrils induced by citric acid (CA) has been reported [66]. At acidic conditions (pH 2), CA induced the crosslinking of WPI fibrils via hydrogen bonds. At neutral conditions (pH 7) and alkaline conditions (pH 10), CA promoted covalent crosslinking, and more disulfide bonds were formed. The formed hybrid hydrogels showed increased firmness and stability of gastric degradation compared to the heat-denatured WPI gel [7]. Munialo et al. [47] found that soy protein, pea protein, and whey protein fibrils could be induced to form gels via glucono-δ-lactone (GDL).

Transglutaminase could chemically crosslink *β*-lg nanofibrils to form a cold-set gel. The hardness of the hybrid gel was higher than that of the pure *β*-lg nanofibrils gel because transglutaminase increased the thickness of the network strands in fibril gels [69].

Wang et al. [70] found that the mechanical properties of *β*-lg nanofibril gels are reinforced upon the diffusion of polysaccharides (low-acetylated gellan gum and κ-carrageenan). The sugar chains diffused inside the mesh and formed high bending rigidity and thicker bundles or an independent network. The rheological and texture properties of sodium alginate/WPF (SA/WPF) hydrogels were enhanced by increasing the concentration of WPF via hydrogen bonding and electrostatic interactions [71]. Moreover, research conducted by Roshanghias et al. [72] has demonstrated that the consistency coefficient of WPF gels was increased by adding CNCs and alginate. The alginate-WPF gel had negative swelling, which can be used for the burst release of drug and bioactive substances. Additionally, Wei et al. [73] reported that stable hydrogels could not be formed by ovotransferrin nanofibrils alone. At the same time, the electrostatic interactions between ovotransferrin fibrils and xanthan gum (XG) might contribute to fabricating stable hydrogels. The gel strength and viscosity of ovotransferrin nanofibril–XG were higher than those of an XG gel. Hybrid hydrogels were made via the reassembly of whey nanofibrils and chitin nanofibrils, and the neutralization of the whey nanofibrils’ isoelectric point charge was the main gelation mechanism [74].

A PEG-lysozyme nanofibril (PEG-LZMF) hydrogel was prepared by a nucleophilic substitution reaction between -NH2 on LZMF and N-hydroxysuccinimide groups on PEG [75]. The generated hydrogel was antibacterial, anti-swelling, injectable, and adhesive. The mechanical properties of the hydrogel were strengthened with the increase in solid contents. Recent studies have shown that the non-electrostatic physical interaction between polyphenol and lysozyme fibrils drives the self-assembly of complex nanofilaments which further form supramolecular hybrid hydrogels [76]. Different polyphenols with different hydrophilicity and hydrophobicity may lead to different strengths of hybrid gels. The polyphenol gallol density and complex ratio with fibrils regulated gel behavior. He et al. [77] showed that the hybrid EGCG- lysozyme fibril gel showed a dense network and porous microstructure, while gallic acid—and chlorogenic acid—lysozyme fibril gel had a loose network and large pores. The EGCG crosslinked lysozyme fibril hydrogel was mainly driven by hydrogen bonding, π-π stacking, and hydrophobic interactions [78]. The loading capacities of EGCG and EGC in the hybrid gels were as high as 4.0 wt%, significantly higher than other delivery systems [79]. This hybrid gel was thermally resistant, antibacterial, and anti-inflammatory. Oral administration of the hybrid gel significantly mitigates colitis and regulates gut microbial dysbiosis in a mouse model. Additionally, lysozyme fibrils, zein, and EGCG have also been employed in hybrid hydrogels [80]. Lysozyme fibrils can perform as the scaffold, while zein nanoparticles can be induced by EGCG and deposited on the surface of the fibrils. When coated with hybrid hydrogels, the storage quality and the shelf-life of beef could be enhanced. Similarly, the gel strength was enhanced via the physical interactions among lysozyme nanofibrils, EGCG, and Fe^3+^. Through further crosslinking with genipin, the microstructure of the hybrid gels became denser, which accounts for the increased gel stiffness [81]. Ji et al. [82] prepared soy protein fibrils-based hydrogel co-encapsulated curcumin (Cur) and (−)-EGCG (UFCE). UFCE gels exhibited better gelling properties and encapsulation efficiency of Cur and EGCG. 

CaCO_3_ nanoparticles (CaNPs) were used as physical crosslinkers, and Ca^2+^ was used as a charge screening ion in the *β*-lg fibrils network to establish and stabilize the network [83]. Compared with the calcium-induced fibril gel, the gel strength of the *β*-lg-CaNPs gel was increased by two orders of magnitude.

### 4.3. Aerogels

Aerogels are highly porous and elastic and formed through freeze-drying or supercritical carbon dioxide-assisted drying. FPN aerogels are materials with a variable structure and functionality suitable for wide-ranging potential applications. *β*-lg fibril aerogels show a low density (≈0.044 g cm^−3^) and high conductivity in diverse applications [84]. Additionally, it has been reported that the aerogel formed by *β*-lg nanofibrils shows obvious brittleness and is easy to disintegrate. Still, its mechanical properties can be improved by introducing polyvinyl alcohol (PVA) and polyethyleneimine [85]. This hybrid aerogel can remove hexavalent chromium from wastewater excellently. Aminosilane-modified *β*-lg and black bean protein fibril-templated aerogels had a promising CO2-capture ability [20]. Furthermore, an aerogel-hydrogel biphase gel (AHB-gel) was prepared via the physical crosslinking between *β*-lg fibrils and PVA [86]. The hybrid gel was a candidate material for wound healing.

## 5. Properties of FPN Gels

### 5.1. Water-Holding Capacity

The WHC of the gel reflects the amount of aqueous phase retained in the protein network. It is very important to product processing and the sensory properties of food [36]. The WHC largely depends on the stiffness, pore size, water distribution, electrostatic interaction, and hydrophobic interaction of the gel. Research has revealed that WPI can produce fibril gels with a homogeneous and dense network at pH values far from their isoelectric point [36]. Meanwhile, when the pH value is next to the isoelectric point, whey proteins form particulate gels with a coarse structure. Therefore, WPI fibril gels with small pores showed a higher WHC than WPI particulate gels with large pores [62,87]. Khalesi et al. [58] found that the WHC value of a long-fibril-containing hydrogel was higher than that of a short- fibril-containing hydrogel, which may be attributed to the more branched and compact microstructure of the gels containing the long fibrils. 

The properties and functions of protein gels depend not only on the crosslinked network structure but also on the state and distribution of water in the gel network. The stronger gels had a higher WHC and the shorter the T_2_ relaxation time in low-field nuclear magnetic resonance. Huyst et al. [88] found that the higher the ovalbumin fibril concentration, the shorter the T_2_ relaxation time.

### 5.2. Mechanical Properties

The mechanical properties of gels are affected by the network structure, network density, and crosslinking mode. Fibrils with an organized β-sheet structure have superior mechanical properties. The non-covalent interactions (hydrophobic and electrostatic interaction) between the side chains of residues further stabilize the fibril structure. Young’s modulus of fibrils is 3.3 ± 0.4 GPa, which is similar to that of silk (1–10 GPa) [89]. The network structure of protein hydrogels is directly related to their mechanical properties. The increase in the β-sheet content in FPN enhances the mechanical strength of FPN gels. 

Texture profile analysis has been used to probe the oral processing of gels. The hardness of the gel reflects the ability of the gel to maintain the interconnection of the internal structure and resist deformation and fracture. Liu et al. [90] found that adding WPF improved the texture properties (the hardness, chewiness, springiness, and cohesiveness) of the gels. Adding NaCl in the range of 0–50 mM reinforced the cohesiveness, gumminess, and chewiness of nanofibril gels [58].

Furthermore, the rheological property of food protein gels is an important quality of the gel and can be used to simulate the mechanical properties of gels [36]. The fibril gel formed at a lower pH had a higher G′ value and better mechanical properties than that formed at a neutral pH [38,39]. The rheological characteristics of FPN gels are associated with the contour length and persistence length of fibrils. The G′ value of the long-fibril-containing hydrogel was higher than that of the short-fibril-containing hydrogel [58]. Adding FPN to gels led to an increase in G′ and G″ values [15,62]. The reason for this result was attributed to the increased entanglements and hydrophobic interactions between fibrils [91].

### 5.3. Thixotropy

Although the cross-β structure of nanofibrils is stable, nanofibrils are affected by environmental conditions and show dynamic changes along with the changes in external conditions, resulting in damage to structural integrity. Therefore, the self-healing property helps the gel prolong its service life. The thixotropy of the gel reversibly breaks under mechanical stress and returns to the original gel state under static conditions, which reflects the self-healing ability of the gel. Mechanistic studies indicate that the non-covalent interaction of side residues, such as hydrophobic and π-π interactions and hydrogen bonding, may contribute to the thixotropicity of nanofibril gels. Bolisetty et al. [48] found that the *β*-lg fibril gel has fast self-healing behavior after completely destroying the structure, which was due to the strong mechanical strength. Yang et al. [10] generated a HEWL nanofibril gel that could be regulated by magnesium ions. In the step-strain oscillatory rheological analysis, this hydrogel network was disrupted and transformed from solid to liquid under high strain (800%) and then recovered under low strain (0.1%). Self-healing hydrogels were also prepared by bovine serum albumin fibril [92]. The gel was damaged by a needle or cut into small pieces. After 24 h, the hydrogel restored the original structure without residual damage. Recently, Yang et al. [93] prepared a dual-nanoengineered DNA dynamic hydrogel via the coassembly of lysozyme nanofibrils and clay nanosheets with DNA strands. The self-healing was experimentally confirmed with multiple stress–strain rheology experiments, and the storage modulus showed instantaneous recovery. The hydrogel was cut and attached along the cutting surface via external force. The results showed that the healing effect was better at 37 °C, and the time needed to realize mechanical recovery was reduced.

*β*-lg nanofibril-CaNPs gel showed pH-triggered self-healing properties. The researchers found that two pieces of *β*-lg nanofibril-CaNPs gel could stick together to form a gel with a clear boundary [83]. This hydrogel was fractured under high strain (1000%) and then recovered under low strain (1%) within 2 h. Because of the excellent thixotropy, nanofibril gels can act as an injectable carrier of drugs and delivery platform for stem cells [94].

### 5.4. Electrical Conductivity

*β*-lg fibrils were assembled into hydrogels via oxidative polymerization of PVA and transformed into aerogels with a three-dimensional porous architecture [84]. The final hybrid aerogels had a high electrical conductivity (≈0.042 S cm^−1^), similar to the electrical conductivity of other reported composite conductive aerogels. Han et al. [84] revealed that *β*-lg fibrils could promote electron transport and be used as a general template to form conductive polymers, which was attributed to their effective surface-to-volume ratio. 

The hybrid aerogels preparation from lysozyme protein nanofibrils, gelatin, and poly(4-(2,3-dihydrothieno[3,4b]-[1,4]dioxin-2-yl-methoxy)-1-butanesulfonic acid had a high conductivity (0.01 S cm^−1^) [95]. Moreover, the aerogels showed a rapid change in electrical current in response to the change in applied forces and can be employed as pressure sensors.

### 5.5. Antibacterial and Antioxidant Activities

Bacterial growth and reproduction or oxidation is one of the main effects during the processing and storage of food. The antibacterial activities of fibrils are much superior to those of natural protein monomers due to the increased β-sheet composition of fibril structures and membrane-disrupting mechanisms [96]. The mechanism behind this mainly includes the destruction of detergent-like membranes and membrane thinning. Firstly, protein nanofibrils are amphiphilic, in which positively charged residues contribute to the interface absorption of microbial anionic membranes. The dense distribution and interaction of nanofibrils in the membrane make the encapsulated lipids peel off from the membrane, thus reducing the interfacial tension of the membrane and eventually leading to membrane fracture. In addition, nanofibrils can seriously distort the membrane shape of microorganisms and even adjust their orientation, resulting in membrane thinning and rupture [97]. In another work, Hu et al. [78] fabricated a PEG crosslinked lysozyme fibril hydrogel, which displayed strong antibacterial activities against Gram-positive and Gram-negative bacteria. They demonstrated that the antibacterial activity of the hydrogel was only derived from lysozyme fibrils. Similarly, hybrid hydrogels composed of lysozyme fibrils, zein, and EGCG could effectively inhibit the growth of microorganisms and the oxidation of lipids [80]. In addition, the antimicrobial peptides released by the hydrolysis of protein monomers in the process of protein fibrillation could improve their antioxidant and antibacterial activity [97].

## 6. Application of FPN Gels

Thanks to their unique physicochemical properties, FPN gels have many potential applications in several fields, including biomedical engineering, water purification and CO_2_ capture, tissue engineering, and energy materials. In this section, we review some of the most promising and exciting examples of where FPN gels have been incorporated into functional devices.

### 6.1. Biomedical Engineering

**Figure 2:**
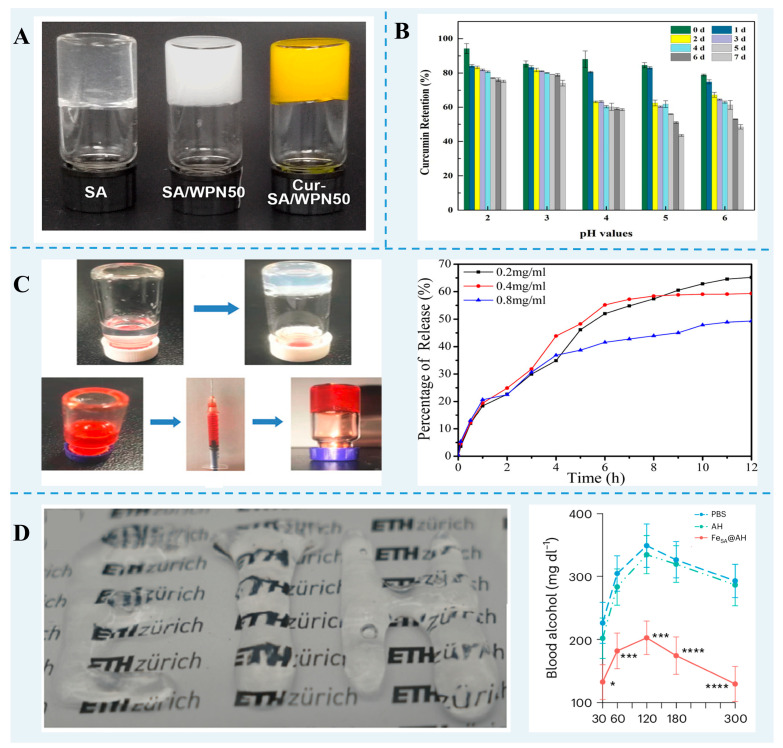
(**A**) The images of SA gel, SA/WPF hybrid gel, and Cur-SA/WPF gel. Reprinted with permission from [71]. Copyright 2023 Elsevier. (**B**) Curcumin retention of WPF-GNP-Cur hydrogel after ultraviolet radiation [59]. (**C**) Injectability of the HEWL fibril hydrogel with doxorubicin entrapment and the release plot of doxorubicin [10]. (**D**) Injectable *β*-lg nanofibril hybrid gel and the mean concentrations of blood alcohol in alcohol-intoxicated mice treated with hybrid gel [94]. * *p* < 0.05, *** *p* < 0.001, **** *p* < 0.0001.

Conventional protein gels have been extensively investigated for bioactive and nutrient delivery. However, the integrity and the sustained release properties of the conventional gel network are not very good. Differentially sourced FPN gels have been applied to increase the loading of bioactives and nutrients and showed controlled release to achieve the targeted treatment (Figure 2). For instance, cold-set SA/WPF hybrid gels bound to curcumin through hydrophobic interactions, van der Waals forces, and hydrogen bonding showed an encapsulation efficiency of 91.6% [71]. Zhu et al. [59] reported that a WPF-GNP hybrid gel exhibited a good encapsulation efficiency and protection effect on the photodegradation of curcumin. An ovotransferrin nanofibril–xanthan gum gel via the electrostatic interaction was more efficient in dihydromyricetin delivery, with a higher dihydromyricetin loading (2 mg/mL) and gastrointestinal release (100%) after gastrointestinal digestion [73]. 

FPN gels have excellent thixotropy and injectability and have great potential in drug delivery. Using doxorubicin as a model drug, an injectable HEWL nanofibril gel showed a 100% loading capacity and a gradually released throughout 12 h [10]. Furthermore, the injection of the *β*-lg nanofibril hybrid gel into alcoholic mice could significantly decrease the level of blood alcohol [94].

Further, both lysozyme fibrils and polyphenols can be used as edible materials with anti-inflammatory properties; thus, oral administration of the lysozyme fibril-EGCG gels could ameliorate colitis in mice by reducing the relative abundances of operational taxonomic units related to colitis, regulate gut microbial dysbiosis and promote the intestinal barrier function [79]. In addition, obesity induced by a high-fat diet was significantly prevented in mice, lipogenesis and pro-inflammatory in liver and adipose tissue were significantly decreased, and lipid metabolism genes were increased.

### 6.2. Water Purification and CO_2_ Capture

**Figure 3:**
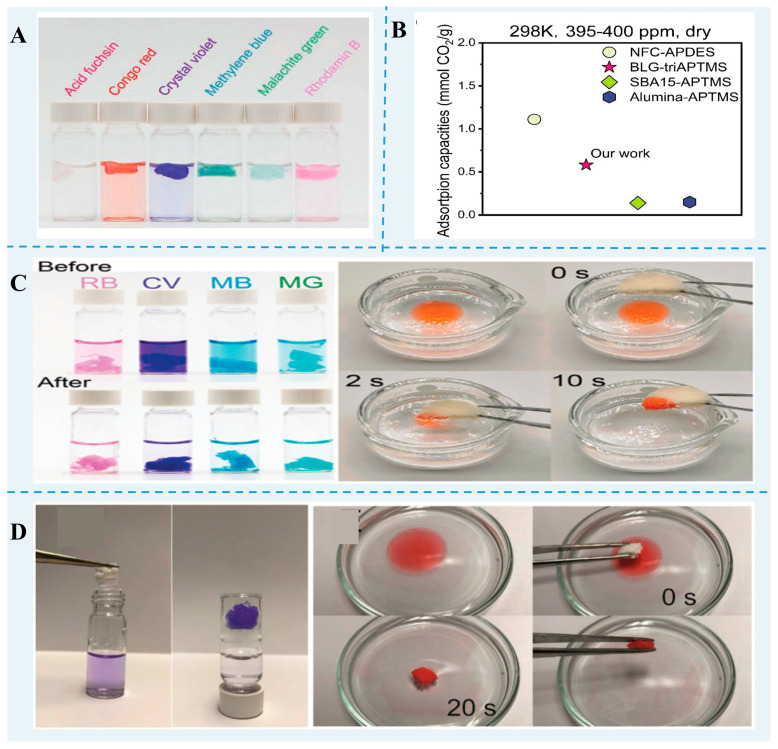
Water purification and CO_2_ capture of FPN gels. (**A**) The removal of dyes from water using *β*-lg nanofibril/zeolitic imidazolate framework-8 hybrid aerogels [101]. (**B**) CO_2_-adsorption capacity of amine-functionalized *β*-lg fibril-templated aerogels [20]. (**C**) Dye- and organic solvent-removal performance of *β*-lg nanofibril and UiO-66-NH_2_ hybrid aerogels [99]. (**D**) The removal of dye and n-hexane from the water via *β*-lg nanofibril aerogels. Reprinted with permission from [100]. Copyright 2020 WILEY.

Toxic pollutants, such as organic pollutants, heavy metals, and CO_2_ emissions, constitute a significant threat to human life. Recently, many biopolymers of food waste and byproducts have been investigated as adsorbents for CO_2_ and toxic pollutants (Figure 3). Protein nanofibrils with cross-β structure have stable mechanical properties. After freeze-drying, aerogels are formed and can be developed as solid adsorbent support. Zhang et al. [42] revealed that nanofibrils could be an efficient solution for water purification due to their high aspect ratio, antibacterial properties, abundant functional groups, and high strength and stability. Peydayesh et al. [98,99] investigated carbon aerogels based on whey and vegetable protein nanofibrils. They found that the protein nanofibril carbon aerogels were able to adsorb much more Au(III), Pt(II), and Fe(III) than whey gels, which may be ascribed to a higher surface area of fibril and the amount of exposed amino acids. Moreover, this aerogel maintained excellent removal efficiency over three continuous regeneration cycles, indicating its remarkable reusability. They also found that *β*-lg nanofibril aerogels have excellent removal efficiencies for Bentazone, Bisphenol A, and Ibuprofen [100]. 

Zhou et al. [20] prepared aminosilane-modified nanofibril-templated aerogels assembled from different food proteins. A *β*-lg nanofibril-templated aerogel with a CO_2_-adsorption capacity of 51.52 mg (1.17 mmol) CO_2_/g at 1 bar CO_2_, was superior to that of a black bean protein and lysozyme fibril-templated aerogel. Furthermore, the hybrid aerogel prepared using a *β*-lg nanofibril and UiO-66-NH_2_ had a greater capacity for CO_2_ adsorption and removal performance in different heavy metals and organic dyes than the pure *β*-lg fibrils aerogel [99].

### 6.3. Tissue Engineering

**Figure 4:**
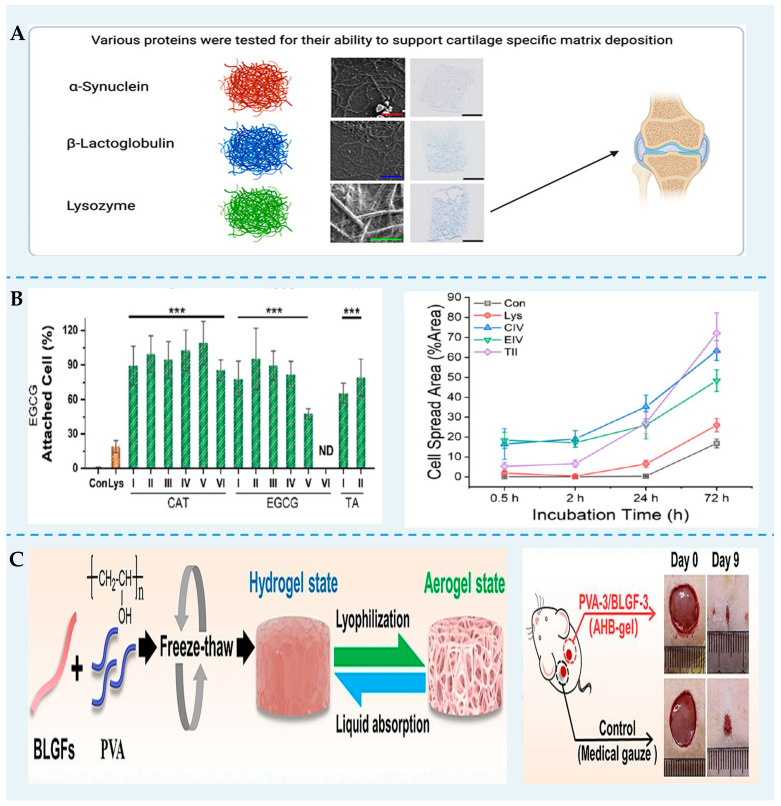
FPN can be used as biomaterials for tissue engineering. (**A**) Different FPNs affect the cellular response and tissue formation [102]. (**B**) The superior cell viability of lysozyme fibrils–polyphenol hybrid hydrogels. Reprinted with permission from [103]. *** *p* < 0.001. Copyright 2023 American Chemical Society. (**C**) The formation of AHB-gel and its application in improving the rate of wound healing [86].

Hydrogels have been discovered as biomaterials for tissue engineering owing to their similar structure and composition to the natural extracellular matrix. Many FPNs are very stiff and have a very slow biodegradation rate because of their structure rich in β-sheets. Thus, FPN can be an effective cell-scaffolding biomaterial (Figure 4). Recent studies have demonstrated that the protein used for self-assembled nanofibrils determines tissue regeneration [102]. Lysozyme, α-synuclein, and *β*-lg supported cartilage formation to a certain extent. However, only lysozyme nanofibrils hydrogels could correctly form collagen bundles. Yan et al. [49] reported that lysozyme nanofibril gels were transparent and self-supporting. Using two- and three-dimensional cell cultures of 3T3 fibroblast cells, it was proved that the gel was biocompatible, promoting cell attachment, spreading, and proliferation. Wu et al. [103] designed lysozyme fibril–polyphenol hydrogels via non-covalent self-assembly. They showed that this hybrid gel was biocompatible and bioadhesive and could be used as a biocompatible scaffold for cellular proliferation and spreading. Wei et al. [104] fabricated nanofibril networks from soy protein using biological and physical crosslinking methods. The fibril scaffolds had higher Young’s moduli (3.9 kPa) than bovine muscle (1.2 to 1.8 kPa) under a 5% strain. Furthermore, C2C12 mouse skeletal myoblasts could proliferate and differentiate on these stable scaffolds without adding cell-adhesion agents. Thus, the soy protein nanofibril gel can be utilized as scaffolding materials for cultivated meat. 

Since the freeze-dried *β*-lg nanofibril aerogel immediately re-disperses in water, Nyström et al. [105] used butane tetracarboxylic acid as a crosslinker to stabilize the scaffolds. These soft, elastic, and water-stable aerogels were suitable for the penetration and permeation of Caco-2 and HT29. Gong et al. [86] found that adding *β*-lg nanofibrils improved the microporous structures, softness, and biocompatibility of the AHB-gel dressings. The AHB-gel dressings could effectively improve the rate of wound healing and accelerate the formation of a new epidermis layer. Moreover, these AHB-gel dressings can be designed differently by combining cytokines or drugs and hybridization with other materials.

### 6.4. Energy Materials

**Figure 5:**
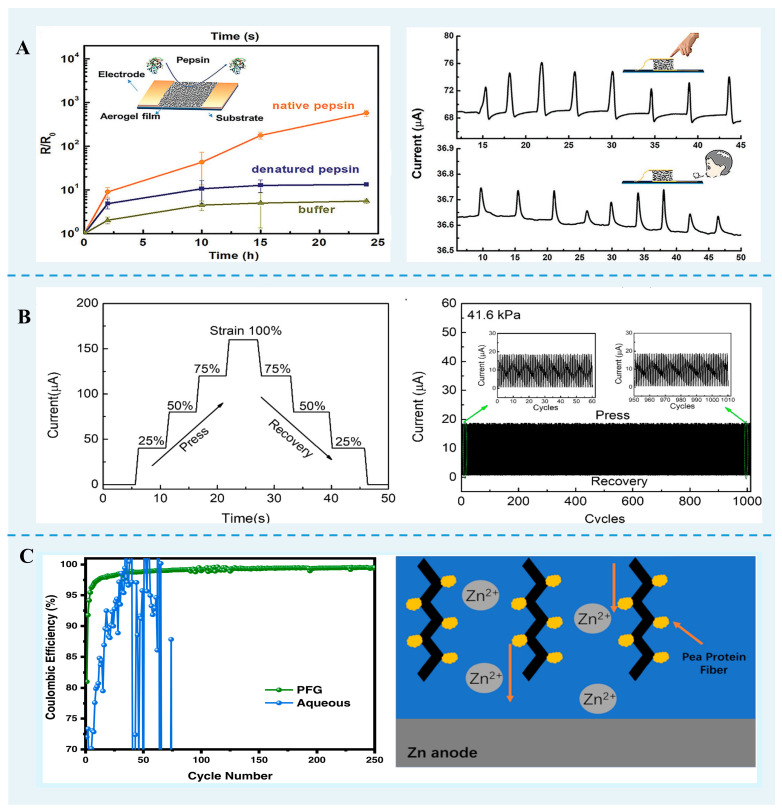
FPN gels are used as energy materials. (**A**) The aerogel film enzyme biosensor. Reprinted with permission from [84]. Copyright 2020, Wiley. (**B**) Current–time curves of lysozyme nanofibril hybrids aerogels [95]. (**C**) Pea protein nanofibril and vinyl alcohol hydrogel enhanced the zinc anode stability. Reprinted with permission from [106]. Copyright 2023 American Chemical Society.

FPN gels also have important applications in energy materials (Figure 5). Flexible and biodegradable hydrogels were made from vinyl alcohol and pea protein nanofibril and used as electrolytes in aqueous zinc-ion batteries. This phenomenon was because of the hydrogen bond network between nanofibril functional groups and water molecules. In addition, the interaction between functional groups on pea protein nanofibrils and Zn^2+^ constructs ion channels for the even migration of Zn^2+^, avoiding dendrite growth [106].

Based on *β*-lg fibrils-PVA hybrid aerogels with high electrical conductivity and compressibility, Han et al. [84] developed pressure sensing to detect the fingertip pressures and air movements of human respiration. Furthermore, these aerogels could be exploited as biosensors to evaluate enzyme activities, such as native pepsin, denatured pepsin, trypsin, and α-chymotrypsin. The electrically conductive hybrids aerogels with elastic properties can be formed from mixtures of lysozyme protein nanofibrils, poly(4-(2,3-dihydrothieno[3,4b]-[1,4]dioxin-2-yl-methoxy)-1-butanesulfonic acid, and gelatin [95]. The sensitivity of this aerogel sensor is 1.80 kPa^−1^, comparable to that developed using conductive aerogels derived from other biomass. Similarly, the hybrid β-lg nanofibril-gold aerogels can be used as a pressure sensor device due to the softness and compressibility of the material [107].

## 7. Conclusions

In this review, we comprehensively reviewed the properties and applications of FPN gels. We focused on the influencing factors of FPN gels and summarized the properties of different FPN gels. In addition, their use in bioactive and nutrient delivery, adsorbents for CO_2_ and toxic pollutants, cell-scaffolding biomaterials, and biosensors were described. The gelation conditions and applications of different FPN gels are summarized in Table 1.

Looking to the future, research on FPN gels should focus on the following aspects: First, more applications only focused on a few proteins, such as WPI, SPI, and HEWL, but less research on other proteins. Extraction of proteins from food waste to fabricate FPN gels could reduce the cost and achieve resource utilization. Recently, sunflower and peanut protein nanofibrils were fabricated to form nanofibril-carbon membranes. The hybrid membranes could remove toxic heavy metal pollutants from contaminated water [25]. Second, despite in vitro and in vivo studies clarifying FPN as a potential safe nutritional ingredient for human health applications [112], their effect on foods and long-term toxicity should also be considered. Additionally, although ultrasound treatment [113], microwave [114], and ohmic heating [115,116], as cost-efficient and safe technologies, have been widely utilized to fabricate nanofibrils, variations in stability and mechanical properties may affect the reproducibility and reliability of FPN gels in practical applications.

## Figures and Tables

**Table 1:** Gelation conditions and application of FPN gels.

Proteins		Fibrillization Conditions	Gelation Conditions	Types	Application	Ref.
milk protein	WPI	pH 2, 90 °C, 24 h	metal ions	hydrogel		[56]
		pH 2, 90 °C, 5 h, 350 rpm	carbohydrate	carbon aerogel	water purification	[108]
		pH 2.0, 85 °C, 5 h	GNP pH 2.0, 85 °C, 3 h	hybrid gel	encapsulate curcumin	[59]
	*β*-lg	pH 2.0, 80 °C, 8 h	sodium alginate	cold-set hydrogel	encapsulate curcumin	[71]
		β-LG/BSA pH 2.0, 80 °C, 8 h	Ca^2+^-induced	cold-set hydrogel	delivery of riboflavin	[67]
		90 °C, pH 2, 5 h	polymerization, freeze–thaw, drying	aerogel	pressure sensing device	[84]
		pH 2, 363 K, 5 h (150 r/min)	mixed with PVA and polyethyleneimine, freeze-dried	hybrid aerogel	water purification	[85]
		90 °C, pH 2, 5 h	mixed with PVA, lyophilization	hybrid gel	wound dressings	[86]
	κ-Casein	pH 8.0, dithiothreitol, 37 °C	dialysis against distilled water, room temperature	hydrogel	drug delivery	[109]
hen egg-white lysozyme		pH 2, 90 °C, 8 h	EGCG-iron; genipin crosslinked	hybrid hydrogel/aerogel		[81]
		pH 2, 90 °C, 8 h	crosslink polyphenols	hybrid hydrogel	antibacterial activity, anti-inflammatory	[77,78,79]
		pH 2, 90 °C, 12 h	crosslink the PEG/gelatin	hybrid hydrogel	sealing and repairing injured tissues	[75]
		pH 2.2, 65 °C, 4 h	crosslink the PEG/gelatin	hybrid hydrogel	drug delivery	[60]
		90 °C, pH 2.0, 8 h	zein and EGCG	hybrid hydrogel	coating of beef	[80]
		25 mM HCl, 80 °C, 24 h	gelatin	hybrid hydrogel	piezoresistive pressure sensor	[95]
		pH 2, 200 mM MgCl_2_, 65 °C, 5 d	pH 7.3, 37 °C, 5–10 min	hydrogel	injectable drug carrier	[10]
		90 °C, pH 2, 350 rpm, 24 h	polyphenols, room temperature, 12 h	hybrid cold-set hydrogel	cell scaffolding	[103]
ovalbumin		78 °C, 22 h, pH 7; trypsin, 37 °C, 48 h		hydrogel		[88]
		pH 3, 90 °C	with EGCG	hybrid hydrogel		[61]
		85 °C, 24 h, pH 2	with resveratrol	hybrid cold-set hydrogel	delivery of resveratrol	[110]
ovotransferrin		pH 2, 90 °C, 26 h	mixed with XG and GDL	hybrid hydrogel	dihydromyricetin deliver	[73]
bovine serum albumin		90 °C, TCEP, 90 min	5% *w*/*v* TCEP, room temperature	hydrogel	cell culture	[92]
soy protein isolate		pH 2.0, 85 °C,12 h	the MTGase induced	hybrid hydrogel		[111]
		pH 2.0, 80 °C, 8 h	EGCG and Cur, 85 °C, 5 min	hybrid hydrogel	co-encapsulation of EGCG/Cur	[82]
		85 °C, pH 2, 20 h	pH 7.5, 37 °C, 24 h, transglutaminase and CaCl_2_ crosslinking	hybrid hydrogel	scaffold for cultivated meat	[104]
		85 °C, 20 h	mixed with CNC,95 °C, 30 min	hybrid hydrogel		[64]
black bean protein		pH 2, 90 °C, 10 h	APTMS-fibril, dried by supercritical CO_2_	aerogel	capture of CO_2_	[20]
pea protein		85 °C, 20 h	GDL induced	cold-set hydrogel		[47]
			vinyl alcohol	hybrid hydrogels	electrolyte of zinc-ion batteries	[106]
lentil protein		90 °C, 0.5–16 h		hydrogel		[38]
kidney bean protein		90 °C, pH 2, stirring at 300 rpm, 10 h	dialyzed for 2 days with NaCl solution	cold-set hydrogel		[46]
faba bean protein		pH 2, sonicated for 30 min, 90 °C		hydrogel		[21]

## Data Availability

No new data were created or analyzed in this study. Data sharing is not applicable to this article.

## Abbreviations

The following abbreviations are used in this manuscript:

FPN	food protein nanofibrils
WPI	whey protein isolate
β-Lactoglobulin	β-lg
SPI	soy protein isolate
WPF	WPI nanofibrils
GNP	gliadin nanoparticles
WHC	water-holding capacity
CNCs	cellulose nanocrystals
EGCG	Epigallocatechin gallate
PEG	poly(ethylene glycol)
HEWL	hen egg-white lysozyme
CA	citric acid
GDL	glucono-δ-lactone
SA	sodium alginate
XG	xanthan gum
Cur	curcumin
PVA	polyvinyl alcohol
AHB-gel	aerogel-hydrogel biphase gel
